# Challenges and opportunities of centring the African voice in disability research

**DOI:** 10.4102/ajod.v11i0.1089

**Published:** 2022-10-31

**Authors:** Lieketseng Y. Ned, Kudakwashe Dube, Leslie Swartz

**Affiliations:** 1 Department of Global Health, Faculty of Medicine and Health Sciences, Centre for Disability and Rehabilitation Sciences, Stellenbosch University, Cape Town, South Africa; 2 Africa Disability Alliance, Pretoria, South Africa; 3 Department of Psychology, Faculty of Arts and Social Sciences, Stellenbosch University, Cape Town, South Africa

**Keywords:** disability, AFRINEAD, politics of voice, ideational space, African Renaissance

## Abstract

In 2020, the African Network of Evidence to Action on Disability (also known as AFRINEAD) hosted its 10th conference in Cape Town. This paper synthesises inputs by the three authors as plenary addresses, particularly focusing on the challenges and opportunities of centring African voices in disability research. Our concern in this article is to engage with the question of exclusion as an issue not just in the everyday lives of people with disabilities but also in the world of ideas – the ideational space. We suggest that a reimagined disability study depends on the centring of African experiences, voices and knowledges. This is especially so as there are African concepts that are not rigorously pursued in research. African Renaissance thinking makes allowance not only for critically reflecting on the historical and contemporary constructs of disability but also for fashioning a higher civilisation in which people with disabilities can exist within society as worthy and valued human beings.

## Background

In 2020, the African Network of Evidence to Action on Disability (also known as AFRINEAD) hosted its 10th conference in Cape Town. Because of the coronavirus disease 2019 (COVID-19) pandemic restrictions, this conference took place virtually. The theme of this conference was ‘Disability unplugged – what really matters to persons with disabilities in Africa’. As part of the proceedings of the conference, one of the opening plenary sessions included three keynote speakers. The keynote speakers briefly talked about issues related to disability research evidence. This paper synthesises these three keynote presentations, particularly focusing on the challenges and opportunities of centring African voices in disability research. Methodologically, it takes the form of a state-of-the-art type of a review, which is a generic literature review. According to Grant and Booth (2009), this type of review tends to report on current matters, it is typically narrative and its analysis focuses on the current state of knowledge and highlights priorities and potential opportunities for future research. The first author transcribed and synthesised all three presentations and highlighted key issues raised across the presentations. The other authors edited and cross-checked the synthesis. The following sections highlight the focus of these integrated keynote addresses, and all three authors agreed on what is to be included in this paper.

The advent of the COVID-19 global pandemic has thrust the often neglected and eschewed subject of disability into the spotlight. The pandemic has brought the vulnerability of all people into focus. While the pandemic has been a universal experience, however, it has led to particular difficult experiences for many people with disabilities. Some of the factors that inform the specific effects of the pandemic on people with disabilities include the increased risk of poor outcomes from the disease and the adverse social impacts of efforts to mitigate the pandemic (Shakespeare Ndagire & Seketi 2021). In addition to this, people with disabilities, who already have greater healthcare needs and are more likely to experience poor health, face significant challenges in terms of accessing healthcare (Ned et al. 2020). And while the pandemic has been noted for the extent to which it decimated national economies, an equally important reflection must be made on the ways in which it crystallised ideas about which lives and bodies matter and, consequently, which ones do not. On the African continent, people with disabilities, in and out of institutional settings, were and are still being treated as an afterthought. The result of this, among other things, has been an increase in the mental health challenges faced by children and young adults with physical and intellectual disabilities (Theis et al. 2021). In addition, the approach to factoring only comorbidities as the key indicator of risk for contracting COVID-19 effectively ignores the interconnectedness between vulnerability to severe illness and death and the environmental and sociospatial barriers that impede on the lives of people with disabilities (Ned et al. 2020). This illustrates the extent to which responses to the pandemic lacked a disability-inclusive approach and, in the process, deepened the existing alienation of people with disabilities.

The pushing of people with disabilities to the margins during the pandemic is an extension of the various ways in which they are systematically omitted from everyday life on the African continent and the Global South more broadly. This is an issue not just in the everyday lives of people with disabilities but also in the world of ideas – what some term the ideational space. Our concern in this article is to engage with the question of this particular exclusion – to make sense not only of how and why people with disabilities’ contributions in the ideational space are largely disregarded – this is particularly so for all indigenous people with disabilities in Africa (Driskill 2010). We shall suggest that a reimagined disability study depends on the centring of African experiences, voices and knowledges. This is especially so as there are African concepts that are not rigorously pursued in research. Research on these concepts and knowledge can produce evidence about their meaning, intent, utility and understanding of disability and humanity itself (Mji et al. 2011; Owusu-Ansah & Mji 2013). The insistence upon a reimagined disability study is thus a quest for the humanisation of people with disabilities in a world that dehumanises and decivilises them.

## The implications of differential exclusion from education

While the right to basic education is guaranteed in the constitutions of many African countries, this right does not seem to translate adequately to people with disabilities. The World Report on Disability (World Health Organization and World Bank 2010) posits that people with disabilities in Africa and in the Global South broadly have been differentially excluded from education when compared with others in their countries. At a global level, people with disabilities in countries of the Global South are significantly more excluded from education when compared to people with disabilities in the wealthier countries of the Global North. This exclusion from the acquisition of knowledge has direct implications for the opportunities for people with disabilities to participate adequately in the production and dissemination of research. People with disabilities are often positioned as the objects of research rather than equal thinkers and knowledge bearers in their own right (Ned 2022). Furthermore, lack of access to education limits the forms of activities that they can engage in as a means of livelihood generation. This reinforces existing prejudices about what kind of work and thinking they can do – prejudices which are persistent in Africa (Wolfe et al. 2013). More than this, the exclusion of people with disabilities from the ideational space could call into question the very legitimacy and validity of the knowledge that is being produced in disability studies (Swartz 2018). If it is true that the insider voice and the personal are critical in the field of disability studies, as argued by Garland-Thomson (2005) and Swartz (2018), then logic should dictate that the exclusion of these voices and persons significantly undermines the research that is being produced. While there is appreciation that insider knowledge has its limitations (Swartz 2014), there is no doubt that it is crucial not only in expanding what we know about disability but also in changing the rules of how we come to know about disability.

There is ample evidence in historical records that scholarship is often used to set parameters for the exclusion and even, sometimes, annihilation of groups of people who are deemed undesirable by society (Ned 2022). Scholarship provides an appeal to authority that may not always be. Ideological and academic justifications for the abuse, dehumanisation and maltreatment of people with disabilities have played a key role in sustaining abusive practices. Swartz (2018:17) emphasises Goodley et al. (2017), who argued that ‘ideas about what constitutes the human and the nonhuman have bodily consequences for people whose bodies or minds do not fit the norm’. Hence, Swartz (2018) notes that it is evident that there are strong links between social exclusion and oppression and the dominant discursive ideas related to essential questions about disability, as well as questions of personhood. For this reason, if people with disabilities in Africa and the Global South at large are to define and reclaim their personhood, the starting point must be in participating in the very development of ideas that shape the spaces they occupy.

## Challenges for disability-related research in Africa

It is indisputable that there is limited disability research from the frame of reference of people in these African societies and the broader Global South (Ned 2022). Although the picture is changing (with this journal being an example), the field of disability studies is still characterised by the dominance of ideas from the Global North to the exclusion of experiences and knowledges of those in the Global South (Ned 2022). Historically, African countries have, in many ways, been portrayed as marginal and irrelevant to the dominant sites of knowledge production in the Global North (Comaroff & Comaroff 2015; eds. Grech & Soldatic 2016; Ned 2022; Owusu-Ansah & Mji 2013; Swartz 2018). In many of these countries, people with disabilities have been subjected to social, economic, political, cultural, epistemic and spatial exclusion. These exclusions reflect and expand exclusions based on race and class, which are embedded in their colonial histories (eds. MacLachlan & Swartz 2009). This is evident in countries like South Africa where, as Ned (2022) puts it, disability research and provision have been historically interlinked with the hierarchical racialised power which has informed issues of access and use. For this reason, attempts to redress the inequalities and inequities in disability knowledges in South Africa and the African continent broadly necessitate engagement with the much broader question of power (Ned 2022). The implications of the dominance of Global North thinking in disability studies have far-reaching consequences. These issues are explored by Connell (2000), who contends that in laying claims to universality, and thus extrapolating Northern discourse to explain African realities, this Global North thinking ignores the realities that people with disabilities in Africa must navigate. Furthermore, this domination facilitates a situation where African thinkers are all too often uncited and unacknowledged, cementing the Eurocentric idea that Africa is not a site with valid and scientific knowledge systems (Ned 2022).

How, then, should the field go forward? In the next section, we suggest one of several paths.

## African Renaissance as a premise for reimagining disability studies in Africa

As we have noted, the field of disability studies is reflective of geopolitical and broader power relations, and there is limited work which centres doing disability research from the lens of African societies (Ned 2022). This is to say, concepts, debates and research strategies from the Global North largely frame research data from the periphery, specifically from Africa. This is deeply problematic, as the majority of people with disabilities worldwide are located in the Global South (Greening 2015). Furthermore, it has been suggested that as many as 40% of Africa’s population may be living with disabilities (Nyangweso 2021). This necessitates the retheorisation of disability studies through the employment of theories embedded in African Renaissance – ‘a political and epistemological lens for understanding and rebelling against imperialism and neocolonial advances in formerly colonised African societies’ (Sesanti 2016 cited in Ned 2022:3). The concept of the African Renaissance has mobilised debates in South Africa, specifically since former president Thabo Mbeki’s time, as evidenced in the New Partnership for Africa’s Development (NEPAD). However, before Mbeki’s time, Cheikh Anta Diop shaped and pioneered the concept, specifically emphasising the need for a political, economic, cultural and epistemic rebirth in the African continent (Diop 1996), upon recognising the way in which Africans have been dehumanised by colonisation and colonialism. Mbeki (1998) later followed Diop’s footsteps and reiterated the African Renaissance idea in his inaugural address as a Chancellor of the (then known as) University of Transkei in Umtata in 1995 and later in various high-level meetings such as the Corporate Council on Africa in Chantilly, Virginia, United States of America, in 1997 and another which was held in September 1998 in Johannesburg, South Africa (Cossa 2009). Such a lens recognises the various ways in which development processes are a key factor to poverty and underdevelopment, which contribute significantly to both the creation and the reproduction of disability (Chouinard 2014). Disability is not only about physical impairment but also about the sociospatial, environmental, cultural and economic constructs that produce and reproduce it. This understanding grounds the work of critical disability and decoloniality scholars who are posing salient questions around whether and how coloniality and the colonial and neocolonial system not only reinforce ableism but also create disability and disabled bodies (Meekosha 2011). This work builds on the contributions of scholars such as Fanon, who, in *The Wretched of the Earth* (Fanon 2005), draws the link between colonialism and mental pathologies in colonised subjects. For example, Fanon notes how treatments of inferiority emanating from both the ableist ideologies and colonial oppression and exclusion play are key factors in the creation of mental illness and a poison towards one’s sense of self (Ned, Kpobi & Ohajunwa 2021).

The legacies of colonialism can be observed not only in apparently high rates of impairment but also in the ways that people with disabilities are treated in contemporary Africa – from being hunted for magic potions and denied access (Tanner 2010) to people with albinism being particularly killed for false beliefs related to get-rich-quick schemes (Ojok & Musenze 2019). The treatment of people with disabilities mimics extractive and demeaning practices which were central to the colonial project. African Renaissance calls for an insistence on a view of Africa and Africanness in which beliefs about disability are integrative and where people with disabilities are given visible roles in society and part of common activities of daily living (Ned 2022). This reflects the African philosophy of *ubuntu*, which locates disability politically within the wider environment and sustainability practices which are integral to participation and inclusion of people with disabilities (Berghs 2017). This is reflected in African proverbs and idioms such as ‘it takes a village to raise a child’. Applied to childhood disability, a sense of belonging may provide a key guard rail against the alienation and victimisation of children with disabilities. Isolation, exclusion and hence maltreatment may be seen to be linked to a Western philosophy of individualism and extractive labour practices, whereby people are measured individually in terms of how they produce for the dominant economy (Ngoetjana 2007). African Renaissance thinking therefore makes allowance not only for critically reflecting on the historical and contemporary constructs of disability but also for fashioning a higher civilisation in which people with disabilities can exist within society as worthy and valued human beings.

## Conclusion

The COVID-19 pandemic has highlighted the necessity of a disability-inclusive approach in responding to structural challenges in Africa. It has demonstrated the ways in which a disabling society reproduces conditions that give rise to disability. This approach must be evidence-based and have at its centre the intellectual labour of those with disabilities. The challenges in disability research demand dedicated critical scholarship and dedicated activism on the part of disability scholars and activists. They also demand great reflection and interrogation. It is important as we move forward to reflect on how, unwittingly, even progressive initiatives like capacity-building may reinforce dominant power relationships and the enforcement of normalcy (Swartz 2018). Sustained reflection is necessary to help us avoid the reproduction and reinforcement of exclusionary practices that confront people with disabilities in Africa. We suggest that African Renaissance ideas may be a more humanising approach; core to this approach, though, is not just an appeal to Africanness but also a commitment to self-reflection and to taking responsibility for our own power and its possible disabling consequences for other Africans.
